# Molecular and Genetic Pathogenesis of Oral Cancer: A Basis for Customized Diagnosis and Treatment

**DOI:** 10.3390/biology14070842

**Published:** 2025-07-10

**Authors:** Leonor Barroso, Pedro Veiga, Joana Barbosa Melo, Isabel Marques Carreira, Ilda Patrícia Ribeiro

**Affiliations:** 1 Maxillofacial Surgery Department, Unidade Local de Saúde de Coimbra, 3000-075 Coimbra, Portugal; leonor.barroso@gmail.com; 2 Institute of Cellular and Molecular Biology, Cytogenetics and Genomics Laboratory, Faculty of Medicine, University of Coimbra, 3000-548 Coimbra, Portugalmmelo@fmed.uc.pt (J.B.M.); icarreira@fmed.uc.pt (I.M.C.); 3 Coimbra Institute for Clinical and Biomedical Research (iCBR) and Center of Investigation on Environment Genetics and Oncobiology (CIMAGO), Faculty of Medicine, University of Coimbra, 3000-548 Coimbra, Portugal; 4 Center for Innovative Biomedicine and Biotechnology (CIBB) and Clinical Academic Center of Coimbra (CACC), University of Coimbra, 3000-548 Coimbra, Portugal

**Keywords:** oral cancer, squamous cell carcinoma, liquid biopsies, signaling pathways, genetic biomarkers, circulating tumor DNA, circulating tumor cells, circulating tumor RNA, extracellular vesicles

## Abstract

Oral cancer, the most common form of head and neck cancer, is worldwide a serious public health problem. Most patients present a locally advanced disease, and face poor prognosis, even with multimodality treatment. They may also develop second primary tumors in the entirety of their upper aerodigestive tract. The most altered signaling pathways are the PI3K/AKT/mTOR, TP53, RB, and the WNT/β-catenin pathways. Genomic and molecular cytogenetic analyses have revealed frequent losses at 3p, 8p, 9p, and 18q, along with gains at 3q, 7p, 8q, and 11q, and several genes frequently affected have been identified, such as *TP53*, *CCND1*, *CTTN*, *CDKN2A*, *EGFR*, *HRAS*, *PI3K*, *ADAM9*, *MGAM*, *SIRPB1*, and *FAT1*, among others. Various epigenetic alterations were also found, such as the global hypomethylation and hypermethylation of *CDKN2A*, *APC*, *MGMT*, *PTEN*, *CDH1*, *TFP12*, *SOX17*, *GATA4*, *ECAD*, *MGMT*, and *DAPK*. Several microRNAs are upregulated in oral cancer, including miR-21, miR-24, miR-31, miR-184, miR-211, miR-221, and miR-222, while others are downregulated, such as miR-203, miR-100, miR-200, miR-133a, miR-133b, miR-138, and miR-375. The knowledge of this molecular pathogenesis has not yet been translated into clinical practice, apart from the use of cetuximab, an EGFR antibody. Oral tumors are also genetically heterogenous and affect several pathways, which means that, due to the continuous evolution of these genetic alterations, a single biopsy is not sufficient to fully evaluate the most adequate molecular targets when more drugs become available. Liquid biopsies, either resorting to circulating tumor cells, extracellular vesicles or cell-free nucleic acids, have the potential to bypass this problem, and have potential prognostic and staging value. We critically review the current knowledge on the molecular, genetic and epigenetic alterations in oral cancer, as well as the applications and challenges of liquid biopsies in its diagnosis, follow-up, and prognostic stratification.

## Simple Summary

Oral cancer is the most common form of head and neck cancer and remains a serious global health concern; it is often diagnosed at advanced stages and is associated with poor prognosis. Treatment is still based on surgery, radiation, and chemotherapy, and despite advances in targeted therapies and immunotherapy, the prognosis is still poor. Multiple genetic and epigenetic alterations have been identified, including changes in key signaling pathways such as PI3K/AKT/mTOR, TP53, and WNT/β-catenin. Genomic studies have revealed frequent chromosomal gains and losses, mutations in several cancer-related genes, and deregulated microRNAs. Despite this growing molecular knowledge, its application in clinical practice is still limited, with cetuximab being one of the few targeted therapies currently used. Due to the genetic heterogeneity of oral tumors and their dynamic evolution, single biopsies may not provide a complete picture. In this context, liquid biopsies offer a promising, less invasive alternative for monitoring disease progression and identifying therapeutic targets. We critically review the current knowledge on the molecular, genetic, and epigenetic alterations in oral cancer, as well as the applications and challenges of liquid biopsies in its diagnosis, follow-up, and prognostic stratification.

## 1. Clinical Aspects of Oral Cancer

### 1.1. Epidemiology

Oral cancer is the most common of head and neck cancers, with an incidence of 389,485 cases each year, with 188,230 deaths [1,2]. Oral cancer is the sixteenth most common cancer worldwide, and in Southeast Asia it is the most frequent among males and third among females [1,3]. Males are 2.43 times more likely to be afflicted than females (5.8 vs. 2.3 per 100,000) [2]. Its incidence has increased globally in the past 30 years [4] and rises exponentially with age, from 0.09–0.13 per 100,000 under the age of 25 years to 14.4–28.8 per 100,000 after the age of 75 years [2,5], with a median age at diagnosis of 66 years [6].

Over 90% of oral cancers arise from the mucosal epithelium and are classified as squamous cell carcinoma. Appearing as an ulcerated mass on any location of the mouth (buccal mucosa, tongue, gum, floor of the mouth, hard palate, retromolar trigone), it should be easy to recognize, but most patients are diagnosed with advanced lesions, often with regional metastasis [7]. Oral cancer may evolve from areas of altered mucosa, with so-called oral potentially malignant disorders (OPMD), but it usually develops from seemingly normal mucosa, most often in patients exposed to known carcinogens.

Smokers have an 8.4-fold increased risk of developing oral cancer, compared to nonsmokers [3,8]. The use of smokeless tobacco (snuff, chewed) is also carcinogenic [9]. Alcohol abuse, even without smoking, increases oral cancer risk [10], but the two carcinogens have a synergistic effect, which might increase the risk of those that smoke and abuse alcohol to 35 times that of abstemious nonsmokers [11]. In India and Southeast Asia, the use of smokeless tobacco combined with betel leaves, areca nuts, and lime, is responsible for the highest incidence of oral cancer worldwide [6,12]. Other factors, such as poor diet, insufficient oral hygiene, and other lifestyle factors appear to be minor risks [13].

In a minority of patients, genetic factors are important: those with Fanconi’s anemia have a 500–700-fold increase in head and neck cancers, mainly in the oral cavity [14]. Other genetic disorders, such as Li Fraumeni Syndrome, Bloom Syndrome, and ataxia–telangiectasia also show an increased incidence of oral cancer [15].

Oral potentially malignant disorders (OPMD) such as leucoplakia, erythroplakia, oral lichen planus, oral lichenoid lesions, oral submucous fibrosis, and proliferative verrucous leukoplakia are “any oral mucosal abnormality that is associated with a statistically increased risk of developing oral cancer” [16,17]. Their prevalence and probability of evolving into oral cancer varies and is difficult to calculate, but it is estimated that 4.47% of the world’s population may harbor one of the OPMD, and the overall risk of malignant transformation was calculated as 7.9% [16,18].

### 1.2. Diagnosis and Treatment

The diagnosis of oral squamous cell carcinoma (OSCC) depends on the histopathological evaluation of incisional or excisional biopsy, usually with routine hematoxylin and eosin staining, with immunohistochemistry employed in poorly differentiated and basaloid lesions. These tumors resemble the stratified epithelium of the oral mucosa, with increased cellular atypia and less squamous differentiation in poorly differentiated tumors [6].

In oral cancer the first treatment is usually surgery (comprising tumor resection, defect reconstruction, and neck dissection) that, apart from initial stages, is followed by radiation therapy or concurrent chemoradiation with platinum salts or, when those are contraindicated, with cetuximab, an EGFR-directed antibody. An inoperable disease is usually treated with chemoradiation.

Metastatic and recurrences where re-operation/re-irradiation is not feasible are addressed with chemotherapy (platinum, taxoids, 5FU), targeted drugs (cetuximab), and immunotherapy (nivolumab, pembrolizumab) [19].

A range of immunotherapeutic strategies is currently under investigation, including immune checkpoint inhibitors, co-stimulatory receptor agonists, antigen-based vaccines, oncolytic virus therapies, and adoptive T-cell transfer approaches [20].

Therefore, identifying reliable and clinically practical predictive and prognostic biomarkers is essential to optimize patient selection for treatment.

### 1.3. Prognosis

The prognosis of OSCC is mostly dependent on clinical stage, with survival rates varying between localized, regional, and distant stages (e.g., 84%, 70%, and 41%, respectively, for tongue cancer) [7,21]. It also varies with the location in the oral cavity, with 91% 5-year survival for the lip, 69% for the tongue, and 53% for the floor of the mouth [21]. The prognosis has improved slightly, mainly in the early stages [12,22], but not as much as expected with the improvement in surgical procedures, radiation technics, and chemotherapy and the introduction of molecular targeted therapies and immunotherapy.

Many surviving patients develop recurrences, distant metastasis, and carry a nearly 20-fold higher risk of developing a second cancer in the following 5 to 10 years [2,23]. Locoregional relapses, metastasis, and second tumors all carry a poor prognosis, which worsens with subsequent adverse events [24,25]. Survivors of head and neck cancer also face an increased risk of mortality from any cause, including other cancers and cardiovascular and pulmonary diseases, presumably in relation with tobacco and alcohol abuse [23], but also from suicide [26]. Patients and their families are burdened with physical and psychosocial sequelae, as the tumor and the treatment affect major functions such as swallowing, chewing, talking, and breathing and may cause facial disfiguration, with a large effect on health-related quality of life [6,27].

## 2. Biological Characteristics of Oral Cancer

### 2.1. Field Cancerization and Intratumor Heterogeneity

The occurrence of several OSCC in the same patient, either simultaneously or sequentially, has been noted for a long time, leading to the concept of “Field Cancerization”.

This phenomenon has been described in other malignancies, such as colon, stomach, esophagus, lung, breast, prostate, skin, and bladder [28,29], but was first observed in oral cancer patients. Even before the understanding of the molecular mechanisms of cancer or the identification of the carcinogenic agents, Slaughter et al. proposed that the buccal epithelium was “preconditioned and irreversibly changed, so that the process towards cancer became inevitable” [30].

This concept of “field cancerization” has been used to describe and explain the presence of a wide area of mucosa affected by pre-malignant diseases, the occurrence of multiple tumors in this altered mucosa, and even distant related tumors in the upper aerodigestive tract [31]. It was demonstrated that the histologically normal mucosa adjacent to the tumor has tumor-associated genetic alterations [28,32,33], and those can be present even in the mucosa on the opposite side from the initial tumor [34,35]. Recent advances, particularly through next-generation sequencing (NGS), have confirmed the phenomenon of field cancerization and revealed significant genetic variability across different regions of the same tumor. Ultra-deep sequencing studies have further elucidated the subclonal architecture and evolutionary dynamics of oral cancer.

More recently, the recognition of the role of stem cells in epithelial homeostasis and the development of cancer stem cells was included in this concept of field cancerization: mutated stem cells, with mutations that confer growth advantages (such as in the *TP53* and *NOTCH* family genes), lead to the expansion of these mutated clones, with the formation of a mutated patch and, afterwards, a mutated field, where the addition of further genetic alterations leads to dysplasia, carcinoma in situ, and invasive and metastasizing carcinoma [28,36,37].

The mutated stem cells can also migrate, either by submucosal spread or by distant implantation, originating discontinuous fields where the progression to a full-fledged tumor can occur, which is made easier if the mutagenic aggression persists, such as the persistence of smoking, causing second primary tumors and explaining some local recurrences when the surgical resection was apparently complete [28,29,38,39].

The continuous evolution of these tumors and fields causes a great genetic heterogeneity, which has been associated with worse outcomes after treatment, with more local relapses, metastasis, and second field tumors occurring among the tumors [40,41] and fields [42] with the highest genetic heterogeneity. So, high intratumor heterogeneity has been associated with poorer clinical outcomes, underscoring its prognostic value [43]. This intratumor heterogeneity will also hamper the choice of personalized targeted therapies, as a single biopsy will not show the full mutational landscape of the cancer [44,45]. The complexity and heterogeneity of tumor cell populations play a critical role in driving tumor evolution. Although some studies have investigated intratumor heterogeneity in OSCC, current data remain limited and insufficient to capture the full extent of this phenomenon. Both intertumor and intratumor heterogeneity can manifest across spatial and temporal dimensions and are reflected at multiple molecular levels, including genetic and epigenetic alterations, metabolic profiles, and post-translational protein modifications. Understanding the molecular evolution of somatic alterations within tumors may ultimately support the development of more individualized treatment strategies.

### 2.2. Molecular Pathogenesis of Oral Cancer

#### 2.2.1. Cell Signaling Pathways and Therapeutic Targets

**Figure 1:**
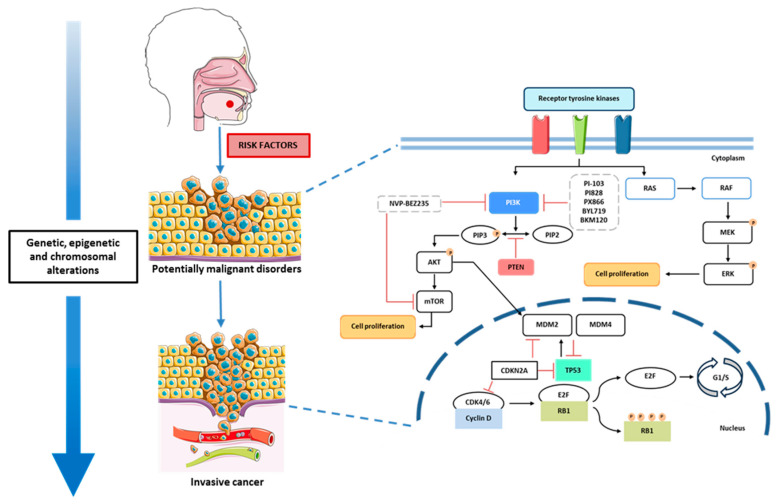
Oral cancer pathogenesis and simplified schematic representation of the PI3K/AKT/mTOR, RB, p53, and MAPK pathways. Receptor tyrosine kinases (RTKs) activate the PI3K and MAPK pathways. PTEN negatively regulates the PI3K pathway, while tumor suppressors TP53 and CDKN2A control the cell cycle by inhibiting the cyclin D-CDK4/6 complex and preventing the activation of RB1-E2F, which is essential for cell cycle progression. Parts of the figure were drawn using Servier Medical Art licensed under a Creative Commons Attribution 3.0 Unported License.

The molecular pathogenesis of oral cancer is complex and involves multiple alterations in various cellular processes that ultimately culminate in the malignant transformation of the oral mucosal cells (Figure 1) [46]. During this process, cells gradually acquire genomic alterations that contribute to tumor development. These alterations may occur in specific genes involved in various cellular pathways, namely the PI3K/AKT/mTOR pathway, MAPK pathway, TP53 pathway, RB pathway, and the WNT/β-catenin pathway, among others (Figure 1) [47].

The PI3K/AKT/mTOR pathway is activated through the binding of growth factors, such as EGFR (epidermal growth factor receptor), to receptor tyrosine kinases (RTKs). Once activated, PI3K phosphorylates phosphatidylinositol-4,5-bisphosphate (PIP2) into phosphatidylinositol-3,4,5-trisphosphate (PIP3). PIP3 then activates the AKT protein, which recruits mTOR, leading to cell proliferation. Additionally, the AKT protein interferes with MDM2 [48,49]. PTEN acts as an antagonist of this pathway by inhibiting the conversion of PIP3 back into PIP2 [48,50].

This cell signaling pathway is involved in crucial processes, including proliferation and survival, angiogenesis, and epithelial–mesenchymal transition (EMT) [50]. The EGFR gene is frequently overexpressed in oral cancer due to amplification, contributing to the activation of this pathway [46]. The overexpression of EGFR, a well-characterized oncogenic driver, has become a useful biomarker. Its presence correlates with aggressive tumor behavior and poorer clinical outcomes, making it not only a prognostic indicator but also a therapeutic target [51].

Several studies have highlighted the therapeutic potential of PI3K inhibitors in the treatment of OSCC. Aggarwal et al. (2019) demonstrated that PI3K inhibitors, such as PI-103, PI828, and PX-866, appear promising for the treatment of oral cancer, exhibiting anti-tumor activity by inhibiting inflammation, blocking the cell cycle, suppressing angiogenesis, and enhancing apoptosis [52]. In another in vitro study conducted by Su et al. (2014), the use of NVP-BEZ235, a dual PI3K and mTOR inhibitor, in conjunction with radiotherapy appeared to overcome radiation resistance during treatment [53]. Further supporting the anti-tumor efficacy of NVP-BEZ235, Hsu et al. (2018) demonstrated that this drug inhibited the proliferation and migration of OSCC cells [54].

Furthermore, Chuang et al. (2021) [55] evaluated the effects of two additional PI3K inhibitors, Alpelisib (BYL719) and Buparlisib (BKM120), and observed a marked reduction in tumor cell growth. However, this inhibitory effect was notably absent in radiation-resistant cell lines, indicating that resistance mechanisms may limit the efficacy of PI3K-targeted therapies in certain contexts [55].

Other targeted therapies, such as cetuximab, a monoclonal antibody that inhibits EGFR, appear to be associated with tumor growth inhibition [56,57]. However, the activation of the PI3K signaling pathway has been associated with resistance to this treatment. In this context, Tsuchihashi et al. (2020) combined the use of Alpelisib with cetuximab, which resulted in a greater inhibition of oral cancer progression compared to the individual administration of these drugs [58].

These findings demonstrate the critical involvement of the PI3K/AKT/mTOR pathway in oral cancer progression, particularly through its regulation of cell proliferation, survival, angiogenesis, and resistance to therapy. The frequent overexpression of EGFR and subsequent activation of this signaling cascade contribute significantly to tumor development and treatment challenges. Targeting this pathway with PI3K inhibitors—such as PI-103, PX-866, NVP-BEZ235, Alpelisib, and Buparlisib—has shown considerable promise in preclinical studies by reducing tumor growth, enhancing apoptosis, and overcoming therapeutic resistance. These studies collectively emphasize the potential of PI3K-targeted therapies in oral cancer.

The mitogen-activated protein kinase (MAPK) pathway (Figure 1) plays a crucial role in regulating cellular proliferation and differentiation. Its activation during tumor development is implicated in key processes such as apoptosis, angiogenesis, and metastasis [59,60]. This signaling cascade, which includes multiple kinases, is activated in over 50% of oral cancer cases [61]. The JNK1 and JNK2 isoforms have been shown to contribute to tumorigenesis through negative crosstalk with the carcinogenic STAT3 signaling pathway [62]. Moreover, the p38α MAPK isoform promotes cancer progression by inducing the production of proinflammatory cytokines such as TNF-α, IL-1β, and IL-6 within the tumor microenvironment [61]. The MAPK pathway is also involved in epithelial–mesenchymal transition (EMT), a critical step in metastasis; specifically, p38 MAPK interacts with the transcription factor Snail, leading to E-cadherin downregulation and enhanced invasiveness in OSCC [61].

Targeting the MAPK signaling pathway has thus emerged as a promising therapeutic strategy in OSCC. Several targeted therapies have been explored to inhibit specific components of this pathway. MEK inhibitors, such as trametinib and selumetinib, have demonstrated efficacy in reducing tumor growth by inhibiting the Ras/MEK/ERK pathway [61]. In a study involving 17 OSCC patients, trametinib treatment resulted in clinical to pathological tumor decline in 53% of cases [63].

The persistent activation of ERK1/2 has been associated with cell cycle dysregulation and tumorigenesis. The inhibition of the ERK/MAPK pathway has been shown to induce G0/G1 cell cycle arrest and promote apoptosis in OSCC cells, showing its potential as a therapeutic target [61].

p53 is a crucial transcription factor responsible for regulating essential cellular processes. This protein binds to DNA and controls the expression of multiple genes. Under normal physiological conditions, p53 activity remains low, as its expression is tightly regulated by its negative regulator, MDM2 [64]. Mutations in the *TP53* gene not only impact DNA repair pathways and apoptosis but also disrupt cell cycle regulation [65]. Alterations in the *TP53* gene are frequently observed in oral cancer and have been associated with a poor prognosis [66,67,68]. This makes *TP53* a potential therapeutic target and demonstrates its potential use as a prognostic biomarker.

Data obtained from The Cancer Genome Atlas indicates a high frequency of *TP53* mutations in tumors involving the tongue and oral cavity [69]. Supporting this, Sandulache et al. (2018) found that *TP53* mutations were particularly prevalent in oral cavity tumors exhibiting extranodal extension during lymphatic metastasis, a condition strongly correlated with decreased survival rates [70]. Similarly, Lin et al. (2024) demonstrated that *TP53* alterations are significantly associated with the presence of positive lymph nodes, further emphasizing the gene’s involvement in tumor aggressiveness and metastatic potential [71]. Several targeted therapies for specific *TP53* gene mutations have been developed and have shown promising results. Chen et al. (2012) demonstrated through in vitro studies that phenethyl isothiocyanate (PEITC), a compound used in chemotherapy with the ability to restore p53 function, reduces cell growth and induces apoptosis in oral cancer cells [72]. These findings were further supported by Yeh et al. (2014), who confirmed that PEITC directly targets p53, reinforcing its promise as a molecularly targeted agent in the treatment of OSCC [73].

Cell cycle regulation is critical in tumor development, and as the *RB1* gene is a key tumor suppressor involved in cell cycle regulation, its inactivation may contribute to uncontrolled cell proliferation [74]. The RB protein functions as a regulator of the G1-S restriction point, inhibiting cell proliferation by binding to the E2 transcription factor (E2F) [75]. A loss of heterozygosity and genetic alterations in *RB1* have been reported in head and neck cancers, including oral cancer [76,77]. Jayasurya et al. (2005) demonstrated that both the RB and p53 pathways are frequently implicated in oral cancer and that alterations in the RB pathway significantly impact patient prognosis [78]. Furthermore, the evaluation of p16 and cyclin D1 expression can identify patient subgroups with lower survival rates. More recently, Berdugo et al. (2021) demonstrated that *RB1* loss serves as a favorable prognostic marker and occurs exclusively in p16-positive patients with oropharyngeal squamous cell carcinoma [79]. Given the critical role of this pathway in carcinogenesis, it represents a potential therapeutic target [80].

**Figure 2:**
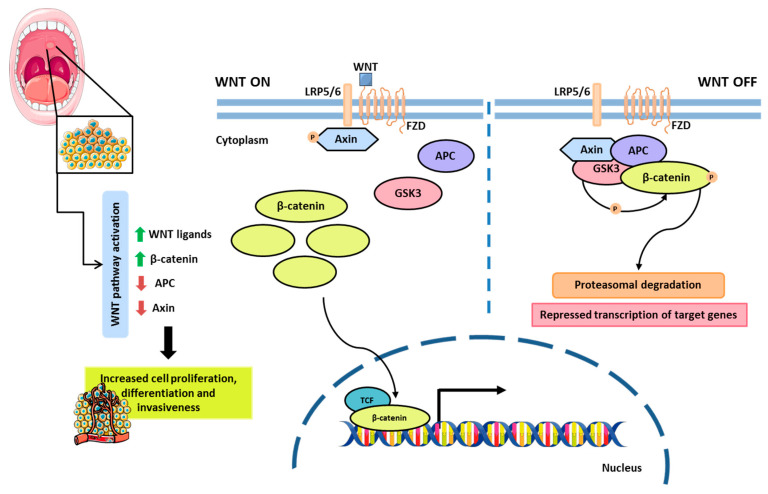
WNT/β-catenin signaling pathway. When WNT ligands bind to the receptors LRP5/6 and FZD (on the left), the phosphorylation and degradation of β-catenin is inhibited. As a result, β-catenin accumulates in the cytoplasm and then in the nucleus, and it interacts with TCF to activate the expression of Wnt-responsive genes. In the absence of WNT ligands (on the right), β-catenin forms a complex with other proteins in the cytoplasm, leading to its degradation via the proteasome. The aberrant activation of the Wnt/β-catenin signaling pathway is primarily driven by β-catenin stabilization and nuclear accumulation, often due to mutations in *APC*, Axin, or β-catenin, or by overexpression of Wnt ligands. Parts of the figure were drawn using Servier Medical Art licensed under a Creative Commons Attribution 3.0 Unported License.

The WNT/β-catenin signaling pathway is involved in various physiological processes, including cell proliferation and differentiation, apoptosis, and tissue homeostasis [81]. This pathway is activated through the binding of extracellular Wnt ligands to membrane receptors, specifically Frizzled and LRP5/6. Upon activation, the protein complex responsible for β-catenin degradation—Axin, APC, and GSK3β—becomes inactivated, allowing β-catenin to accumulate in the cytoplasm and subsequently translocate to the nucleus. Once in the nucleus, β-catenin interacts with transcription factors from the TCF/LEF family, promoting the expression of target genes (Figure 2). In the absence of Wnt ligands, the Axin, APC, and GSK3β complex remains active, leading to the phosphorylation of β-catenin and its subsequent proteasomal degradation, thereby preventing aberrant activation of Wnt signaling [81,82].

During oral cancer carcinogenesis, mutations in genes associated with the WNT/β-catenin signaling pathway frequently occur, resulting in altered gene expression and disrupted protein function. Inactivating mutations in *APC* or Axin, direct mutations in β-catenin, or the overexpression of Wnt ligands can lead to the aberrant activation of this pathway, contributing to tumorigenesis (Figure 2) [83]. Functional studies using animal models have detected nuclear β-catenin accumulation in oral dysplasia samples [84]. Corroborating these findings, Reyes et al. (2020) reported increased β-catenin accumulation in biopsy samples from patients with oral dysplasia [83].

More recently, Campolo et al. (2024) [85] demonstrated that the use of GSK343, a selective EZH2 inhibitor, may represent a promising therapeutic strategy for OSCC. This compound has been shown to affect WNT signaling and regulate the expression of key inflammatory markers involved in OSCC progression [85]. Collectively, these studies emphasize the importance of targeting interconnected molecular pathways in oral cancer to improve prognostic accuracy and treatment efficacy.

#### 2.2.2. Genetic Mechanisms in Oral Cancer

The transition from normal oral epithelium to invasive carcinoma is a multistep process and follows a series of histopathological and molecular phases. Oral cancer development is influenced by factors such as tobacco use, alcohol consumption, and human papillomavirus (HPV) infection. Certain genetic conditions can also increase the risk of development [3].

Risk factors can induce molecular changes related to oral cancer carcinogenesis. The molecular mechanisms involve chromosomal abnormalities, genomic alterations, and epigenetic changes, namely involving oncogenes, tumor suppressor genes, and DNA repair genes [3,86]. Understanding these genetic changes is crucial for developing targeted therapies and improving patient outcomes.

Genomic and molecular cytogenetic analyses have revealed frequent losses at 3p, 8p, 9p, and 18q, along with gains at 3q, 7p, 8q, and 11q [87,88,89]. The amplification of the 11q13 region, which includes genes such as *CCND1* and *CTTN*, has been identified as one of the most common genetic events in OSCC [87,88,89]. The loss of 9p21, encompassing the *CDKN2A* gene, is another frequent alteration related to cell cycle dysregulation and increased malignancy [88,89,90]. The amplification of the EGF receptor (*EGFR*) contributes to cell proliferation and is also a frequent event in OSCC [88,89,90]. Several sequencing and microarray technologies have identified key oncogenic drivers, including EGFR, HRAS, and PI3K pathway components, which are altered in more than 60% of OSCC cases [89]. Regarding the *TP53* gene, sequencing analysis revealed that this gene is also one of the most mutated in OSCC [89,91].

Ultra high-density array CGH studies have identified frequent amplifications at 8p11.23 (*ADAM9*), 7q34 (*MGAM*), and 20p13 (*SIRPB1*), with corresponding overexpression at the mRNA level [87]. The *FAT1* gene is also frequently mutated in oral cancer, influencing the cell cycle and DNA repair [89,92]. Despite extensive genomic research, clinical outcomes in oral cancer have shown only modest improvement. One of the major challenges lies in the sheer volume and complexity of data generated by omics platforms, which often hinders their integration into routine clinical practice. To overcome this, further studies are needed that explore the interplay between genomic and epigenetic alterations and their influence on therapy response. However, both intratumoral and inter-patient heterogeneity continue to pose significant obstacles to generalizing these findings, as genetic and epigenetic profiles can vary markedly within a single tumor and across different individuals. This variability contributes to inconsistent therapeutic responses and disease progression. Consequently, most current treatment guidelines still rely predominantly on clinical and pathological staging rather than molecular profiling [19], highlighting a persistent gap between molecular research and its translation into clinical practice. Bridging this gap will require more robust and targeted translational studies.

#### 2.2.3. Epigenetic Mechanisms in Oral Cancer

The molecular pathogenesis of oral cancer goes beyond genomic alterations, encompassing significant epigenetic changes. These changes involve heritable yet reversible modifications in gene expression without alterations to the DNA sequence, contributing to tumorigenesis [93,94].

The most frequently studied epigenetic mechanisms in cancer include DNA methylation, histone modifications, and non-coding RNAs. DNA methylation, which is mediated by DNA methyltransferases (DNMTs), plays a fundamental role in regulating gene expression and maintaining genomic stability. However, in tumor cells, the normal patterns of DNA methylation are disrupted, resulting in altered gene expression that promotes cell proliferation. Specifically, tumor cells exhibit the global hypomethylation of the genome alongside the hypermethylation of specific regions [93].

Global hypomethylation contributes to chromosomal instability and the activation of proto-oncogenes, both of which drive cancer progression. On the contrary, the hypermethylation of CpG islands within the promoter regions of certain genes leads to the silencing of tumor suppressor genes. Many of these silenced genes are involved in critical pathways such as DNA repair, and their inactivation further facilitates cell proliferation and tumorigenesis [93,95]. In oral cancer, the genes *CDKN2A*, *APC*, *MGMT*, *PTEN*, and *CDH1* are frequently hypermethylated. These genes are associated with various cell signaling pathways, including cell cycle regulation and DNA repair processes [96]. Global hypomethylation is also observed during carcinogenesis, resulting from alterations in the *DNMT1* gene, which is responsible for maintaining DNA methylation homeostasis [97].

Towle et al. (2013) reported that genes involved in the WNT and MAPK pathways exhibit aberrant DNA methylation [98]. Kim et al. (2019) identified frequent hypermethylation in the genes *TFP12*, *SOX17*, and *GATA4*, while Dvojakovska et al. (2018) observed methylation in *ECAD*, *MGMT*, *DAPK*, and *CDKN2A* [99,100].

Histone modifications play a crucial role in the development and progression of oral cancer by influencing gene expression patterns associated with tumor growth, metastasis, and resistance to therapies. These modifications, which include methylation, acetylation, phosphorylation, and ubiquitination, alter the chromatin structure, making DNA either more accessible or restricted to the transcriptional machinery [101]. Histone deacetylases (HDACs) lead to gene silencing. In oral cancer, increased levels of HDACs are related to the repression of important tumor suppressor genes such as *CDKN1A* and *CDH1*. These alterations also potentiate the epithelial–mesenchymal transition (EMT), contributing to cell invasion and metastasis [102]. The efficacy of HDACs inhibitors have shown promising results in several studies regarding various types of cancer, such as breast cancer [103], colon cancer [104], prostate cancer [105], hepatocellular carcinoma [106], and head and neck cancer [107]. HDAC inhibitors have demonstrated antiproliferative effects, induction of apoptosis, cell cycle arrest, and inhibition of angiogenesis and metastasis. These agents also show potential in sensitizing cancer cells to conventional therapies like chemotherapy and radiotherapy. Ongoing research continues to explore HDACi in combination therapies, aiming to enhance their efficacy while minimizing adverse effects, thus offering a promising avenue for improving outcomes in patients with oral cancer. HDAC inhibitors (HDACis), such as valproic acid (VPA) and apicidin, have emerged as promising therapeutic strategies due to their ability to modulate epigenetic dysregulation. VPA, a Class I and IIa HDACi, has demonstrated chemopreventive effects in individuals with high-risk oral dysplasia by altering HDAC gene expression and reactivating tumor suppressor pathways [108]. Similarly, apicidin, a cyclic peptide HDACi, selectively inhibits HDAC8 and has been shown to suppress OSCC cell growth in vitro and in vivo, inducing apoptosis and autophagy, using animal models [109]. Ahn et al. (2011) [110] also had similar results using human oral squamous cell carcinoma cell lines. In this study, apicidin had an antiproliferative effect, inducing cell cycle arrest and apoptosis which triggers the autophagy in OSCC cells [110]. These findings are corroborated by large-scale analyses from The Cancer Genome Atlas (TCGA) and other proteomic datasets, which reveal the upregulation of HDACs in head and neck squamous cell carcinomas and show their prognostic and therapeutic relevance [111]. Together, these findings support the growing interest in HDAC-targeted therapies in the management of OSCC. Also, targeting histone-modifying enzymes may help reverse aberrant epigenetic states, making histone modifications a promising area for therapeutic intervention in oral cancer management.

Beyond genomic characterization, numerous studies have shown the significance of non-coding RNAs in regulating gene expression and their potential involvement in tumor development [112]. These biomolecules can function either as oncogenes or tumor suppressors, influencing tumor progression and development.

MicroRNAs, small single-stranded RNA molecules, play a critical role in regulating the expression of various genes. They are also crucial in processes such as cell division, differentiation, angiogenesis, migration, and apoptosis. Multiple studies have demonstrated the importance of these microRNAs as diagnostic and prognostic biomarkers in various cancer types [113]. In oral cancer, several microRNAs are upregulated compared to samples from healthy individuals, including miR-21, miR-24, miR-31, miR-184, miR-211, miR-221, and miR-222. Other microRNAs are downregulated, such as miR-203, miR-100, miR-200, miR-133a, miR-133b, miR-138, and miR-375. These microRNAs are involved in cell proliferation, metastasis, invasion, and therapy resistance, and their dysregulation contributes to tumor development and progression [114]. Many of these microRNAs exhibit oncogenic potential, acting as oncogenic miRs that inhibit the expression of tumor suppressor genes [114].

Another class of non-coding RNAs extensively studied in oncology is long non-coding RNAs (lncRNAs). These molecules are longer than 200 nucleotides and are involved in the regulation of gene expression. lncRNAs can also interfere with various molecular processes such as splicing, transcription, cell signaling pathways, and mRNA stability [115]. The dysregulation of lncRNAs has been reported in several cancer types [48,115]. In oral cancer, the expression of various lncRNAs is associated with the presence of metastases and poor overall survival. Besides contributing to cell proliferation, migration, and reduced apoptosis, lncRNAs are also involved in epithelial–mesenchymal transition (EMT), as several lncRNAs appear to promote this process [115,116].

In addition to these widely studied non-coding RNAs, small nucleolar RNAs (snoRNAs), circular RNAs (circRNAs), and PIWI-interacting RNAs (piRNAs) have shown evidence of involvement in the development and progression of oral cancer [115,117,118,119]. Therefore, these classes of non-coding RNAs may be valuable targets for further research given their potential.

Advances in OSCC research have led to the continuous identification of novel prognostic biomarkers, reflecting the inherent complexity and heterogeneity of these tumors. Despite these promising developments, the validation of such biomarkers in large patient cohorts remains limited, hindered by the high costs associated with high-throughput technologies and the absence of standardized prioritization frameworks. Furthermore, the successful translation of these biomarkers into clinical practice requires not only improved accessibility to molecular testing but also substantial investments in technological infrastructure and the training of healthcare professionals across all levels of care.

Advancing the clinical application of molecular findings in oral cancer will require a shift toward more holistic and multidisciplinary research approaches.

## 3. Liquid Biopsy in Oral Cancer

Liquid biopsies are a non-invasive method for diagnosis, monitoring, and patient stratification of various cancer types. Unlike traditional biopsies, which require direct tumor tissue excision, liquid biopsy analyzes biological fluids such as blood, saliva, and urine. This approach enables the detection of genetic alterations or tumor-specific biomarkers [48,120]. Additionally, liquid biopsy offers advantages in addressing tumor heterogeneity, as the molecular markers analyzed are expected to originate from multiple tumor regions, making them more representative compared to conventional tissue biopsies [121].

Currently, liquid biopsies have been approved by the FDA (Food and Drug Administration) and EMA (European Medicines Agency) for clinical use in several cancer types, including lung, colorectal, and prostate cancer [122,123]. However, despite its potential, further studies are required to validate and implement liquid biopsy in oral cancer [121,124]. Moreover, liquid biopsies should be used as a complementary tool alongside tissue biopsies and histopathological analysis to improve tumor characterization, facilitate patient monitoring, and assess treatment response [124].

Different methods can be utilized for analyzing tumor components in biofluids. PCR-based techniques—including qPCR, RT-qPCR, and ddPCR—are highly sensitive and specific but are limited to analyzing a restricted number of genetic regions. In contrast, NGS and microarray technologies provide broader and more comprehensive analysis, although they are more expensive and require more complex data interpretation [125,126].

The molecular landscape of oral cancer is marked by extensive genetic and epigenetic heterogeneity, involving mutations in key oncogenes, dysregulation of signaling pathways, and chromatin remodeling by histone modifications and DNA methylation. These dynamic alterations pose significant challenges for conventional tissue biopsies, which often fail to capture the full spectrum of tumor evolution and subclonal architecture. In this context, liquid biopsy emerges as a powerful, minimally invasive approach that enables the real-time monitoring of tumor-specific molecular alterations. The dynamic and heterogeneous molecular alterations driving OSCC pathogenesis, such as *TP53*, *PIK3CA*, and *EGFR* mutations, epigenetic silencing of *CDKN2A* and *MGMT*, and dysregulation of non-coding RNAs, can be effectively monitored using liquid biopsy approaches. These biomarkers provide real-time assessment of therapeutic resistance mechanisms and tumor recurrence. As such, liquid biopsy directly influences the molecular hallmarks of oral carcinogenesis, establishing a clinically actionable link between tumor biology and non-invasive diagnostics.

### 3.1. Circulating Tumor Cells

Circulating tumor cells (CTCs) are tumor cells that invade the circulatory and lymphatic systems after detaching from the primary tumor. These cells play a crucial role in metastasis and have garnered significant attention in cancer research due to their potential applications in diagnosis, prognosis, and treatment monitoring [127].

Several methods have been developed for CTC isolation, including size-based techniques, immunomagnetic assays, and microfluidic platforms [48,128]. In 2004, the US Food and Drug Administration (FDA) approved the first CTC isolation system—CellSearch^®^—which captures CTCs based on the expression of epithelial markers such as EpCAM. However, a major limitation of this method is its inability to detect CTCs that lack EpCAM expression, particularly those undergoing epithelial–mesenchymal transition [129].

Recent findings further highlight the clinical relevance of CTC analysis. Geng et al. (2024) reported that patients in remission or with stable disease exhibited significantly lower CTC counts after treatment compared to baseline levels [130]. Khandare et al. (2020) observed a positive correlation between CTC count and cancer stage, noting that patients with early stage OSCC had fewer CTCs, while those with advanced-stage disease exhibited significantly higher CTC levels [131]. Similarly, Qayyumi et al. (2022) reported high sensitivity, specificity, and accuracy in CTC detection [132]. Their study also revealed a correlation between CTC levels and adverse clinicopathological factors. Zhang et al. (2021) analyzed peripheral blood CTCs in OSCC patients and found an important clinical value in the diagnosis, screening, progression, and evaluation of metastasis [133]. These results show the potential of CTCs as a biomarker for disease progression and treatment response.

The intratumoral heterogeneity and epithelial–mesenchymal transition processes observed in oral cancer facilitate the dissemination of circulating tumor cells into peripheral blood. These CTCs serve as viable biomarkers that carry the cellular and phenotypic diversity of the primary tumor, including EMT-related markers and metastatic potential, making them valuable tools for early detection and prognosis.

### 3.2. Cell-Free Nucleic Acids

Cell-free DNA (cfDNA) consists of DNA fragments released into the bloodstream through apoptosis, necrosis, or active cellular secretion. In oncology, cfDNA analysis has emerged as a promising non-invasive approach for molecular tumor characterization and dynamic disease monitoring [134]. In cancer patients, a significant fraction of cfDNA originates from tumor cells, known as circulating tumor DNA (ctDNA). This ctDNA carries distinct genetic and epigenetic alterations, which can be utilized for patient stratification, disease monitoring, assessing treatment response, and predicting prognosis [135]. Tumor-specific mutations, such as point mutations, insertions, and deletions, are present in ctDNA but absent in normal cfDNA. Detection methods include targeted sequencing and digital PCR. For instance, the use of NGS has been optimized for ctDNA detection, providing information about genomic alterations in cancer patients [136]. Fragmentation profiles also differ: ctDNA tends to be shorter or more fragmented than cfDNA [137]. Additionally, ctDNA often displays cancer-specific DNA methylation patterns, distinguishable from normal cfDNA through methylation-specific assays [138].

However, the implementation of cfDNA analysis in clinical settings presents challenges. The low concentration of cfDNA in plasma, its fragmented nature, and susceptibility to chemical damage can complicate detection and analysis. Therefore, it requires highly sensitive and specific techniques [139].

In oral cancer, elevated plasma cfDNA levels have been observed in OSCC patients compared to healthy controls, with higher concentrations correlating with larger tumor sizes, cervical lymph node metastasis, and advanced disease stages. Increased cfDNA levels are associated with a poor prognosis, suggesting its utility in both diagnosis and prognosis [140]. Beyond plasma, salivary cfDNA analysis has emerged as a promising diagnostic tool in oral cancer [141,142,143,144]. Rapado-González et al. (2022) detected differences between cfDNA fragment concentrations and integrity in saliva of OSCC patients and healthy individuals [144]. Ahmed et al. (2024) demonstrated that 82% of OSCC patients had detectable tumor-specific mutations in their saliva, irrespective of tumor stage or location [141].

For instance, frequent mutations in *TP53*, *PIK3CA*, and *EGFR*—well-characterized drivers of oral cancer—can be detected by NGS in biofluids [145], providing actionable insights into tumor biology and potential resistance mechanisms to targeted therapies, such as cetuximab. Beyond genetic mutations, epigenetic modifications such as DNA methylation have also emerged as critical factors in OSCC detection. Hypermethylation of tumor suppressor genes, such as *RASSF1A* and *DAPK1*, has been reported in saliva samples of head and neck cancer patients and can distinguish early stage disease from healthy controls [143]. These findings suggest that combining mutation analysis with epigenetic profiling could further enhance the accuracy of liquid biopsies in OSCC. The methylation analysis of tumor suppressor genes such as *CDKN2A* and *MGMT* has also been applied in circulating cfDNA from saliva and plasma, highlighting their potential as non-invasive epigenetic biomarkers for early detection and disease monitoring through liquid biopsy [146].

Despite its potential, salivary cfDNA analysis still faces limitations due to contamination with genomic DNA from oral epithelial cells and bacteria. Moreover, the standardization of cfDNA collection and analysis methods remains a challenge [143]. Future research should focus on improving the sensitivity of assays, optimizing and standardizing sample processing protocols [141].

Cell-free RNA (cfRNA), including messenger RNA (mRNA) and non-coding RNAs such as microRNAs (miRNAs) and long non-coding RNAs (lncRNAs), has emerged as a valuable biomarker in OSCC. Unlike cfDNA, cfRNA reflects active gene expression changes and provides insights into tumor dynamics in real time [143].

Differential expression levels of miRNAs and lncRNAs have been reported in the serum, plasma, or saliva of OSCC patients [147,148,149,150]. MacLellan et al. (2012) focused on the differential expression of miRNAs in serum from patients with oral potentially malignant disorders, identifying several miRNAs with strong diagnostic potential [147]. Similarly, Zahran et al. (2015) [148] explored salivary miRNAs demonstrating their ability to distinguish between oral squamous cell carcinoma and precancerous lesions. Also, miRNA-184 provided good diagnostic value [148]. Several upregulated microRNAs such as miR-21, miR-24, miR-31, and miR-221, have been detected in the plasma, serum, and saliva of OSCC patients. These microRNAs can be used as diagnostic and prognostic biomarkers since they are related to tumor progression and resistance to apoptosis [151].

Regarding lncRNAs analysis, Tang et al. (2013) showed that saliva had a detectable amount of lncRNAs [152]. This study revealed that some lncRNAs are differentially expressed in the saliva of OSCC, such as HOTAIR, and it had the ability to distinguish OSCC metastatic patients from primary cancer controls. On the other hand, MALAT-1 had no significant difference between metastatic and nonmetastatic tissue, despite the ability to be detected in saliva.

Fan et al. (2020) showed that LOC284454 was significantly upregulated in the serum of patients with oral cancer, having a good clinical diagnostic value [150]. Differences in the expression of several lncRNAs were also detected by Jia et al. (2021) in the plasma of OSCC patients [149]. They showed that different lncRNAs were expressed in distinct stages of OSCC and that their expression shifted with disease progression. These results demonstrate the capacity of cfRNA analysis for the early diagnosis and staging of OSCC.

Although liquid biopsy represents a promising tool for patient stratification and monitoring, its clinical application is limited by a lack of large-scale validation. To enable its integration into standard care, there is a need for multicenter clinical trials involving large patient cohorts, as well as the development of standardized protocols to ensure the reproducibility and reliability of results. Few biomarkers identified through liquid biopsy approaches have been rigorously tested in diverse patient populations, which is an obstacle to the clinical translation. However, given the extensive intratumor heterogeneity and dynamic molecular evolution of OSCC, liquid biopsy presents a valuable non-invasive method for capturing the real-time genetic and epigenetic landscape of these tumors.

### 3.3. Extracellular Vesicles

Extracellular vesicles (EVs) are small important structures enclosed by a lipid membrane and secreted by cells into bodily fluids. They play a crucial role in intercellular communication by transporting biomolecules such as proteins, lipids, and nucleic acids [153]. EVs can be categorized regarding their size or cellular origin. These mainly include exosomes, microvesicles, large oncosomes, and apoptotic bodies [154]. The variety of cellular information that these structures carry and their stable nature makes them a promising candidate for non-invasive disease monitoring.

The main challenge when studying EVs is related to the difficulty in isolating these structures since they are prone to contamination. There are several methods used in EVs isolation based on their physical and chemical properties. These methods include centrifugation, size-based isolation, affinity-based isolation, precipitation, and microfluidic techniques [153,154,155].

EVs secreted by tumor cells are believed to provide real-time information of the tumor microenvironment since they represent their cell of origin. These tumor-derived EVs can contribute to metastasis, angiogenesis, and resistance to chemotherapy [153,156]. Due to their involvement in disease development and progression they can be important cancer biomarkers. In the context of oral cancer, EVs have gained attention due to their involvement in tumor progression and immune modulation [157]. EVs can promote tumor progression by altering the tumor microenvironment [158,159]. Momen-Heravi and Bala (2018) demonstrated that EVs can carry oncogenic microRNAs and when taken up by monocytes after co-culture can cause the activation of the NF-κB pathway, promoting a proinflammatory and pro-tumorigenic environment [160].

Exosomes can influence the transforming growth factor–β (TGF-β) pathway, contributing to disease progression and resistance to treatment [161]. Dickman et al. (2017) found that EVs could induce angiogenesis by regulating the TGF-β receptor and enhance its expression on the recipient cells [162]. Bano et al. (2023) [163] showed that salivary exosomes could distinguish tobacco consumers from nonsmokers and oral cancer patients. The difference between the size and concentrations of exosomes in the smokers’ group can indicate changes in cell secretions related to the cell transformation [163].

Several serum or plasma exosomal microRNAs are upregulated in oral cancer, demonstrating their potential as biomarkers or therapeutic targets [164]. Targeting these specific microRNAs, it may be possible to develop more personalized and effective therapies that can disrupt key pathways involved in cancer growth, metastasis, and resistance to treatment. The ability to detect these microRNAs non-invasively through blood or saliva further enhances their potential as accessible, cost-effective tools for monitoring oral cancer.

Despite the promising potential, isolating these vesicles remains a challenge due to their size and complexity. Further studies are needed to refine and standardize protocols regarding EVs isolation and analysis.

## 4. Conclusions

Given the frequent late-stage diagnosis of OSCC and its high recurrence rates, the identification of reliable biomarkers—both for guiding therapeutic decision-making and for monitoring treatment response—is crucial to improving clinical outcomes. A deeper understanding of tumor biology enables the development of highly targeted therapies, allowing clinicians to enhance treatment efficacy while reducing the risk of adverse effects. However, the complexity and heterogeneity of oral cancer, along with its resistance to conventional and experimental therapies, continues to hinder substantial improvements in treatment efficacy and patient survival.

In this review, we have explored recent breakthroughs in the field of oral cancer, highlighting advancements in the understanding of its molecular pathogenesis and technological progress in the identification of diagnostic and prognostic biomarkers. Additionally, we have discussed the persistent challenges in early diagnosis, targeted treatment options, tumor recurrence, and patient stratification, as well as potential future research directions that could pave the way for more effective and personalized therapeutic strategies.

The application of targeted therapies has been hindered by the redundancy of the various disturbed pathways, and the great heterogeneity and permanent genetic evolution of these neoplasms; therefore, a combination of targeted therapies may offer a more effective approach than single-agent therapies. In this context, the integration of predictive biomarkers into patient management, particularly using minimally invasive tools such as liquid biopsies, represents a promising strategy to achieve more precise and personalized treatment approaches, ultimately leading to improved survival and quality of life.

cfDNA, cfRNA, lncRNAs, and microRNAs analysis in saliva and plasma offer a promising, non-invasive approach for OSCC detection, prognosis, and treatment monitoring and tailoring. While further validation in large-scale clinical trials is needed, current evidence strongly supports its integration into precision oncology strategies. In the future, as novel therapies become available, molecular and genetic biomarker testing will be mandatory in treatment selection.

As the metastatic processes and the role of the immune system in cancer control are better understood, it seems plausible that liquid biopsies might be able to single out the patients with nodal metastasis, avoiding cervical dissections in patients without them, where the removal of healthy lymph nodes is useless and potentially deleterious.

Other promising applications are the follow-up of patients with OPMD, allowing for an early diagnosis of the malignant transformation that often is clinically unapparent, and even the screening of high-risk populations, such as heavy smokers and betel users.

Recently, assays for circulating tumor components have emerged as promising tools for enhancing post-treatment surveillance. So, liquid biopsies, before and sequentially during treatment, should become a standard test, helping to choose the initial treatment, detect relapses, metastasis, and second primary tumors, and eventually change the targeted therapy when molecular evolution becomes probable.

## Data Availability

Not applicable.

## Abbreviations

The following abbreviations are used in this manuscript:

cfDNA	Cell-free DNA
cfRNA	Cell-free RNA
CGH	Comparative Genomic Hybridization
CTCs	Circulating Tumor Cells
ctDNA	Circulating Tumor DNA
DNMTs	DNA methyltransferases
EMA	European Medicines Agency
EMT	Epithelial–mesenchymal transition
EVs	Extracellular Vesicles
FDA	Food and Drug Administration
HDACs	Histone deacetylases
HDACi	Histone deacetylases inhibitors
HPV	Human papillomavirus
NGS	Next-generation sequencing
OPMD	Oral potentially malignant disorders
OSCC	Oral squamous cell carcinoma
PEITC	Phenethyl isothiocyanate
